# Diagnostic aids in the screening of oral cancer

**DOI:** 10.1186/1758-3284-1-5

**Published:** 2009-01-30

**Authors:** Stefano Fedele

**Affiliations:** 1 Oral Medicine Unit, Division of Maxillofacial, Diagnostic, Medical and Surgical Sciences, UCL Eastman Dental Institute, 256 Gray's Inn Road, London WC1X 8LD, UK

## Abstract

The World Health Organization has clearly indentified prevention and early detection as major objectives in the control of the oral cancer burden worldwide. At the present time, screening of oral cancer and its pre-invasive intra-epithelial stages, as well as its early detection, is still largely based on visual examination of the mouth. There is strong available evidence to suggest that visual inspection of the oral mucosa is effective in reducing mortality from oral cancer in individuals exposed to risk factors. Simple visual examination, however, is well known to be limited by subjective interpretation and by the potential, albeit rare, occurrence of dysplasia and early OSCC within areas of normal-looking oral mucosa. As a consequence, adjunctive techniques have been suggested to increase our ability to differentiate between benign abnormalities and dysplastic/malignant changes as well as to identify areas of dysplasia/early OSCC that are not visible to naked eye. These include the use of toluidine blue, brush biopsy, chemiluminescence and tissue autofluorescence. The present paper reviews the evidence supporting the efficacy of the aforementioned techniques in improving the identification of dysplastic/malignant changes of the oral mucosa. We conclude that available studies have shown promising results, but strong evidence to support the use of oral cancer diagnostic aids is still lacking. Further research with clear objectives, well-defined population cohorts, and sound methodology is strongly required.

## Introduction

Cancer of the head and neck (H&N cancer), including all oral, laryngeal and pharyngeal sites, is the sixth most common cancer, accounting for about 643 000 new cases annually [1]. About 40% of head and neck malignancies are known to be squamous cell carcinomas arising in the oral cavity [2]. Oral cancer is largely related to lifestyle, with major risk factors being tobacco and alcohol misuse. In addition to smoking, the use of smokeless tobacco has been strongly linked to oral cancer [2].

Five-year survival of oral cancer varies from 81% for patients with localized disease to 42% for those with regional disease and to 17% if distant metastases are present [3]. Generally, according to late-stage diagnosis, fewer than 50% of patients with oral and pharyngeal cancers survive more than 5 years. This rate has remained disappointingly low and relatively constant during the last few decades [2-4].

Treatment of oral cancer often produces dysfunction and distortions in speech, mastication and swallowing, and dental health. It can also affect the patient's ability to interact socially, hence it must be considered among the most debilitating and disfiguring of all cancers [4-6].

It is well established that virtually all oral squamous cell carcinomas (OSCCs) are preceded by visible changes in the oral mucosa, usually by way of white (leukoplakia) and red patches (erythroplakia) [2-6]. In addition, there are other inflammatory disorders of the oral mucosa such as lichen planus, submucous fibrosis and perhaps oral fibrosis due to systemic sclerosis that have been associated with an increased risk of OSCC development [7-10]. It is believed that identification and monitoring of these potentially malignant lesions and conditions allows clinicians to detect and treat early intraepithelial stages of oral carcinogenesis, for example mild, moderate or severe dysplasia and carcinoma in situ, all of which generally precede the development of invasive OSCC [2,4,11]. The prognostic implications of diagnosis and treatment of these early intra-epithelial stages of oral carcinogenesis are highly significant due to high survival rates of early OSCC [11].

The International Agency for Research on Cancer (IARC) and the World Health Organization (WHO) have recently stressed that we can reduce a third of a predicted 15 million cancer cases in the future and more effectively manage another third by planning effective cancer control and screening strategies [12,13]. The major focus of these strategies is preventable cancers, such as those associated with tobacco smoking and infection, responsible for 43% of all cancer deaths in 2000, that is 2.7 million fatalities [2,13]. Oral cancer is among the malignancies that would best benefit from this approach. It affects an area of the body that is easy to access for clinical inspection, is preceded by long-lasting mucosal changes, and has preventable risk factors [2].

Nevertheless, most OSCC are currently detected at a late stage (III or IV) [2,4-6,14] and the overall percentage of oral cancers that are localized when diagnosed is very similar to that of colon cancers (36%), even though the mucosa of the colon requires endoscopic examination for evaluation [5].

Several studies have attempted to clarify which are the factors behind the diagnostic delay of OSCC and why figures concerning prevention and early detection of oral cancer have remained disappointingly constant over recent decades. Lack of awareness in the public of the signs, symptoms, and risk factors for oral cancer, as well as a disappointing absence of prevention and early detection by health-care providers, are both believed to be responsible for the diagnostic delay [5,14]. For example, at present time it appears that pelvic examination and Pap smears are more acceptable than looking in the mouth, a view shared by both patients and physicians, this culminating in a significant portion of early oral lesions being missed by patients and/or health care professionals [15].

In 2005 Sankaranarayanan and co-workers reported the first solid evidence that periodic examination of the oral cavity can reduce mortality from oral cancer in high-risk individuals [16]. Their 9-year screening study reported a significant 32% reduction in mortality in high-risk individuals in the intervention group (42% when only male tobacco/alcohol users are considered), suggesting that oral visual screening in high-risk patients could prevent about 40,000 deaths from oral cancer worldwide [16,17].

Further to visual oral examination with the support normal (incandescent) light, a variety of commercial diagnostic aids and adjunctive techniques have been introduced [18,19]. These supposedly can assist in the detection of early cancerous mucosal changes that can be occult to visual inspection and/or to assess the biologic potential of clinically abnormal mucosal lesions [18,19]. The latter aspect is of particular relevance as only a small percentage of mucosal abnormalities (e.g. potentially malignant disorders) are progressive or become malignant and simple visual examination cannot discriminate between these lesions and their non-progressive counterparts. Moreover effective visual aids would be of great benefit in detecting a small, but clinically significant, percentage of dysplasias or early OSCC that are reported to occur within mucosa that appears clinically normal by visual inspection alone [20]. There remains, however, little evidence to support the effectiveness of these adjunctive techniques. The aim of this paper is to review current evidence regarding available diagnostic aids of early detection of oral cancer and provide a critical analysis of their effectiveness.

### Brush biopsy

The oral brush biopsy, also known as OralCDx Brush Test system, consists of a method of collecting a trans-epithelial sample of cells from a mucosal lesion with representation of the superficial, intermediate and parabasal/basal layers of the epithelium [21-24]. This test was specifically designed to investigate mucosal abnormalities that would otherwise not be subjected to biopsy because of low-risk clinical features [21-24]. A specially designed brush is the non-lacerational device used for epithelial cell collection and samples are eventually fixed onto a glass slide, stained with a modified Papanicolaou test and analyzed microscopically via a computer-based imaging system. Results are reported as "positive" or "atypical" when cellular morphology is highly suspicious for epithelial dysplasia or carcinoma or when abnormal epithelial changes are of uncertain diagnostic significance respectively. Results are defined as negative when no abnormalities can be found. The test is considered an intermediate diagnostic step as a scalpel biopsy must follow when an abnormal result is reported (atypical or positive).

Several studies have been performed in an attempt to test the sensitivity and specificity of brush biopsy in detecting dysplasia or OSCC [21-29]. However, inconsistencies and potential bias of these studies have been reported by several authors [18,27,28]. In the majority of studies for example, scalpel biopsy was performed after brush biopsy of lesions with high-risk clinical features, but not after brush biopsy of innocuously-looking lesions [18]. This is believed to alter results regarding sensitivity and specificity of the test in the clinical context where accuracy is much needed (diagnosis of those lesions that appear innocuous and would otherwise not be biopsied) and supports the criticism an intermediate non-diagnostic test would be superfluous when clinical features are highly suspicious for dysplasia/OSCC and a biopsy has to be performed anyway [18,27,28].

Indeed current data show that OralCDx's cytologic test is highly sensitive and specific in detecting dysplastic changes in high-risk mucosal lesions (due to clinical findings suggestive of malignancy), but when used in a low-risk population with benign-appearing oral epithelial lesions, the accuracy is reduced and the rate of false-positive findings increases [18].

Further rigorous studies are needed to investigate the sensitivity and specificity of brush biopsy in detecting in low-risk populations with clinically innocuous lesions.

### Toluidine blue staining

Toluidine blue (TB), also known as tolonium chloride, is a vital dye that is believed to stain nucleic acids. Hence, it has been used for many years as an aid to the identification of clinically occult mucosal abnormalities and as a useful way of demarcating the extent of a potentially malignant lesion prior to excision [30-35]. Several studies on TB have been performed in the past years but the majority of them present significant limitations and methodological biases [18,19]. These have been reviewed in detail by Lingen and coworkers and include (i) absence of randomized controlled trials, (ii) absence of histological diagnosis as a gold standard, (iii) and variability in methods of application [18].

Analysis of current evidence suggests that TB is good at detecting carcinomas, but its sensitivity in detecting dysplasias is significantly lower [18]. Furthermore, there remain a high percentage of false positive stains which impairs its use in primary care settings as a valid screening mean [18,19]. In addition, controversy exists regarding the subjective interpretation of mucosal staining and criteria for positive results (e.g. dark royal blue versus pale blue staining) [18].

At present, TB is best used by experienced clinicians as an adjunct to clinical examination in the evaluation of the biologic potential of potentially malignant oral lesions [18,19]. A recent study [36] showed that TB preferentially stained visible lesions with high risk molecular patterns and predicted risk and outcome in cases where little to no microscopic evidence of dysplasia was present. To date, however, research has not been extended to determine whether TB can identify and predict the risk of progression for epithelial abnormalities that cannot be seen with the naked eye [18,19].

### Chemiluminescence

Clinical inspection of oral mucosa with the aid of chemiluminescent blue/white light was recently suggested to improve the identification of mucosal abnormalities with respect to the use of normal incandescent light [18,19,37-41].

The relevant technology (ViziLite system – Zila Pharmaceuticals, Phoenix, AZ), involves the use of an oral rinse with a 1% acetic acid solution for 1 minute followed by the examination of the oral mucosa under diffuse chemiluminescent blue/white light (wavelength of 490 to 510 nm). The theory behind this technique is that the acetic acid removes the glycoprotein barrier and slightly desiccates the oral mucosa, the abnormal cells of the mucosa then absorbing and reflecting the blue/white light in a different way with respect to normal cells [18,19,37-41]. Hence normal mucosa appears blue, whereas abnormal mucosal areas reflect the light (due to higher nuclear/cytoplasmic ratio of epithelial cells) and appear more aceto-white with brighter, sharper and more distinct margins [37-41].

More recently, the ViziLite system was modified in order to include the use of TB [42] and a new chemiluminescence device (MicroLux DL) was introduced.

Several studies have been performed with the Vizilite system with the attempt to demonstrate its efficacy in to enhance the identification of mucosal abnormalities [37-42]. It should be highlighted that no study has demonstrated that the chemiluminescence can help in differentiating dysplasia/carcinoma from benign lesions [18,19]. Hence, the majority of studies have investigated how chemiluminescence enhances subjective clinical evaluation of intra-oral lesions including brightness, sharpness and texture with respect to routine clinical examination [18]. As these parameters are highly subjective, it is not surprising that results have been contradictory [18,19]. Whilst some authors report that this technique can improve the detection of intra-oral abnormalities (regardless their nature), other reported that the overall detection rate was not significantly improved and the chemiluminescent light produced reflections that made visualization even more difficult than with incandescent light [18,19,37-42]. Furthermore, the majority of the studies are limited by methodological flaws such as lack of histopathological diagnosis or clear objectives (screening device versus case-finding device) [18]. Some studies suggest that chemiluminescence may help identifying occult lesions that cannot be seen with incandescent light but this, however, is not supported by any strong evidence [18,19].

### Tissue Fluorescence Imaging

Tissue autofluorescence has been used in the screening and diagnosis of precancers and early cancer of the lung, uterine cervix, skin and, more recently, of the oral cavity [43]. The concept behind tissue autoflorescence is that changes in the structure (e.g., hyperkeratosis, hyperchromatin and increased cellular/nuclear pleomorphism) and metabolism (e.g. concentration of flavin adenine dinucleotide [FAD] and nicotinamide adenine dinucleotide [NADH]) of the epithelium, as well as changes of the sub-epithelial stroma (e.g. composition of collagen matrix and elastin), alter their interaction with light [18,43]. Specifically, these epithelial and stromal changes can alter the distribution of tissue fluorophores and as a consequence the way they emit fluorescence after stimulation with intense blue excitation (400 to 460 nm) light, a process defined autoflorescence. The autoflorescence signal is finally visualized directly by a human observer. With regards to the oral cavity, normal oral mucosa emits a pale green autofluorescence when viewed through the instrument handpiece whilst abnormal tissue exhibits decreased autofluorescence and appears darker with respect to the surrounding healthy tissue. Autoflorescence technology for inspection of the oral mucosa has been developed by LED Medical Diagnostics Inc. in partnership with the British Columbia Cancer Agency and is marketed as VELscope system [18,19,43].

Several studies have investigated the effectiveness of the VELscope system as an adjunct to visual examination for (i) improving the distinction between between normal and abnormal tissues (both benign and malignant malignant changes), (ii) differentiating between benign and dysplatic/malignant changes, (iii) and identifying dysplastic/malignant lesions (or lesion's margins) that are not visible to the naked eye under white light [18,19,43-46]. Overall the quality of available studies is significantly higher than that of studies upon chemiluminescence and TB as the technology's sensitivity and specificity was compared to gold standard (histopathology) in all patients studied [18,19,43-46]. With regard to the first aspect, autofluorescence imaging of the oral mucosa has been reported to possibly improve lesions' contrast and therefore increase the ability to distinguish between mucosal lesions and healthy mucosa, although further research on different patients population is needed [43]. The ability of autofluorescence to differentiate between different lesion types has been investigated in a few studies and overall the technique seems to show high sensitivity, but low specificity [43]. However, the VELscope system seems to be very promising due to its ability and effectiveness in identifying lesions and lesions' margins that are occult to visual examination under white light [18,19,43-46]. Using histology as the gold standard, VELscope demonstrated high sensitivity and specificity in identifying areas of dysplasia and cancers that extended beyond the clinically evident tumors [18,19,43-46]. A direct clinical application consists of assessing lesion margins in patients with potentially malignant oral disorders therefore improving surgical management [47,48].

However, it should be highlighted that these results are from case series and case reports rather than clinical trials and that no published studies have assessed the VELscope system as a diagnostic adjunct in screening lower-risk populations (e.g. without a history of dysplasia/OSCC) or in patients seen by primary care providers [18,19,43-48].

### Tissue Fluorescence Spectroscopy

In addition to visual autofluorescence, a technique called autofluorescence spectroscopy has been recently tested in oral oncology research [18,19,43,49]. The autofluorescence spectroscopy system consists of a small optical fiber that produces various excitation wavelengths and a spectrograph that receives and records on a computer and analyzes, via a dedicated software, the spectra of reflected fluorescence from the tissue [18,19,43,49]. This technique has the clear advantage of eliminating the subjective interpretation of tissue fluorescence changes. However, the downside is that more variables (e.g. combination of wavelengths, methodology of fluorescence analysis etc) have to be tested and considered and this has led to controversial and often unclear results [18,19,43,49]. Overall, autofluorescence spectroscopy seems to be very accurate for distinguishing lesions from healthy oral mucosa, with high sensitivity and specificity, especially when malignant tumors are compared to healthy mucosa. However, the ability of the technique to distinguish and classify different types of lesion has been reported to be low [18,19,43,49]. Moreover autofluorescence spectroscopy is for practical reasons not suitable to detect new lesions or to demarcate large lesions as the optical fiber can sample only a small mucosal area [18,19,43,49]. This limits the use of spectroscopy to the evaluation of a well defined small mucosal lesion that has been already identified through visual inspection, with the attempt to clarify its benign or (pre)malignant nature. Further research is needed to support this clinical application of autofluorescence spectroscopy.

## Conclusion

The World Health Organization has clearly indentified prevention and early detection as the major targets in the battle to control the oral cancer burden worldwide [2,50]. Prevention and early detection of OSCC and its pre-invasive intra-epithelial stages is still largely based on visual examination of the mouth, although a variety of molecular techniques have been tested and are likely to represent the ultimate goal of oral cancer research [51]. A 9-year randomized controlled trial has shown that screening *via *visual examination of the oral mucosa under white light is effective in reducing mortality in individuals exposed to risk factors. Simple visual examination, however, is well known to be limited by subjective interpretation and by the potential, albeit rare, occurrence of dysplasia and early OSCC within areas of normal-looking oral mucosa. As a consequence, adjunctive techniques have been suggested to increase our ability to differentiate between benign abnormalities and dysplastic/malignant changes as well as identify areas of dysplasia/early OSCC that are not visible to naked eye. Chemiluminescence and autofluorescence are two relatively new techniques that have been investigated with variable results. Available studies have shown promising results, but strong clear evidence to support their effectiveness is still lacking. Major limitations include analysis of small sample sizes, lack of methodologically sound clinical trials, insufficient use of histologic and molecular mapping of optically altered mucosa, need of more detailed analysis of factors rather than cancer that can affect the optical qualities of the oral mucosa (e.g. inflammation, previous chemo- or radio-therapy), and also direct comparison with other detection methods.

Toluidine Blue has been used by clinicians for many years, yet a clear demonstration of TB indications, limitations, as well as strong evidence from methodologically sound clinical trials is still lacking. Brush biopsy is another example of promising novel diagnostic technique that unfortunately has not been supported by robust evidence. Clinical trials have been performed but in the majority of cases results should be read with caution due to biases, variations in research objectives and methodological inconsistencies.

At present, the utilization of these techniques in clinical practice is largely anedoctal and is principally directed to help experienced clinicians at improving their ability to detect dysplasia and early OSCC in high-risk individuals attending secondary and tertiary centers. Moreover, experienced surgeons use some of the described optic aids to improve the identification of a lesion's margins and extensions in the operatory setting, although it is not know then impact these techniques have on a patient's survival and risk of disease recurrence.

Further research with clear objectives, well-defined population cohorts and sound methodology is required before supporting the extensive use of oral cancer diagnostic aids in both primary and specialty settings.

## Competing interests

The author declares that they have no competing interests.
